# Stimulated Biosynthesis of an C10-Deoxy Heptaene NPP B2 *via* Regulatory Genes Overexpression in *Pseudonocardia autotrophica*

**DOI:** 10.3389/fmicb.2020.00019

**Published:** 2020-01-24

**Authors:** Heung-Soon Park, Hye-Jin Kim, Chi-Young Han, Hee-Ju Nah, Si-Sun Choi, Eung-Soo Kim

**Affiliations:** Department of Biological Engineering, Inha University, Incheon, South Korea

**Keywords:** *Pseudonocardia autotrophica*, polyene, regulatory genes, hemolytic toxicity, antifungal activity

## Abstract

Polyene macrolides, such as nystatin A1, amphotericin B, and NPP A1, belong to a large family of valuable antifungal polyketide compounds that are typically produced by soil actinomycetes. Previously, NPP B1, a novel NPP A1 derivative harboring a heptaene core structure, was generated by introducing two amino acid substitutions in the putative NADPH-binding motif of the enoyl reductase domain in module 5 of the NPP A1 polyketide synthase in *Pseudonocardia autotrophica*. This derivative showed superior antifungal activity to NPP A1. In this study, another novel derivative called NPP B2 was developed, which lacks a hydroxyl group at the C10 position by site-specific gene disruption of the P450 hydroxylase NppL. To stimulate the extremely low expression of the NPP B2 biosynthetic pathway genes, the 32-kb NPP-specific regulatory gene cluster was overexpressed *via* site-specific chromosomal integration. The extra copy of the six NPP-specific regulatory genes led to a significant increase in the NPP B2 yield from 0.19 to 7.67 mg/L, which is the highest level of NPP B2 production ever achieved by the *P. autotrophica* strain. Subsequent *in vitro* antifungal activity and toxicity studies indicated that NPP B2 exhibited similar antifungal activity but significantly lower hemolytic toxicity than NPP B1. These results suggest that an NPP biosynthetic pathway refactoring and overexpression of its pathway-specific regulatory genes is an efficient approach to stimulating the production of an extremely low-level metabolite, such as NPP B2 in a pathway-engineered rare actinomycete strain.

## Introduction

**Figure 1:**
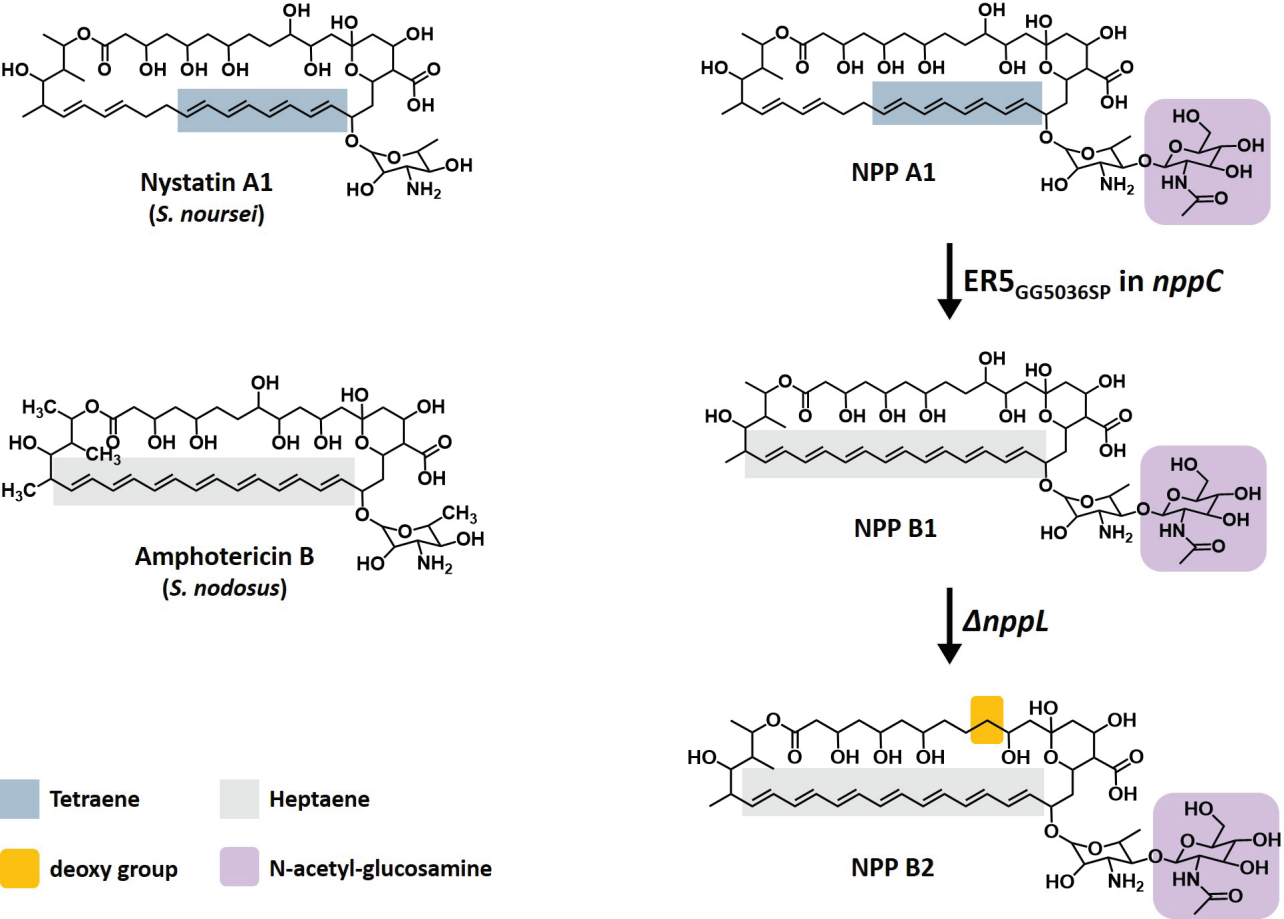
Structure of polyene macrolides. **Nystatin A1**, a typical tetraene polyene macrolide produced by *Streptomyces noursei.* **Amphotericin B**, the most potent polyene macrolide produced by *Streptomyces nodosus.* **Nystatin-like *Pseudonocardia* polyene (NPP) A1**, a disaccharide-containing tetraene polyene macrolide produced by *Pseudonocardia autotrophica.* **NPP B1**, a disaccharide-containing heptaene polyene macrolide produced by *P. autotrophica* ER5 _GG5036SP_ mutant. **NPP B2**, C10-deoxy NPP B1 produced by inactivation of the *nppL* gene in *P*. *autotrophica* ER5 _GG5036SP_ mutant.

More than 45% of the bioactive compounds discovered from microbial secondary metabolites are derived from actinomycetes (Bu et al., 2019). A significant number of these secondary metabolites produced by actinomycetes were utilized further as the lead compounds in the field of medicine as clinically important anticancer, antibiotic, anti-inflammatory, antiviral, antiparasitic, and antioxidant drugs (Bérdy, 2005; Subramani and Aalbersberg, 2012; Manivasagan et al., 2014; Abdelmohsen et al., 2015). Among them are polyene macrolide antibiotics, such as nystatin, amphotericin, candicidin, and pimaricin, which are potent antifungal compounds that are comprised typically of a polyketide core macrolactone ring with about 20–40 carbon atoms, including 3–8 conjugated double bonds (Figure 1; Caffrey et al., 2016). The major antifungal mechanism of these polyene antibiotics is believed to be the formation of ion channels *via* fungal ergosterol binding that mediates the leakage of cellular K^+^ and Mg^2+^, which leads to the death of fungal cells (Bolard, 1986; Neumann et al., 2016).

Over the last several decades, complete polyene biosynthetic gene clusters from nystatin, amphotericin, pimaricin, and candicidin have been isolated and characterized (Aparicio et al., 1999, 2003; Zotchev et al., 2000; Caffrey et al., 2001). Polyene compounds are biosynthesized typically by a giant enzyme complex called polyketide synthase (PKS), followed by further modification of the core macrolide ring by post-PKS modification enzymes, including P450 hydroxylases and glycosyltransferases (Kim et al., 2015). Subsequent attempts to generate a range of derivatives of these antifungal polyene macrolides were pursued to enhance the antifungal activity and/or reduce their intrinsic toxicities through both semisynthetic and biosynthetic pathway refactoring approaches (Byrne et al., 2003; Nedal et al., 2007; Silveira and Husain, 2007; Caffrey et al., 2016). A heptaene version of nystatin A1 called S44HP displayed considerably higher antifungal activity than the original tetraene nystatin A1, which is comparable to that of amphotericin B (Bruheim et al., 2004). Mannosyl-8-deoxy-amphotericins produced by inactivation of the *amphL* gene and introduction of the pIJ02567-*nypY* construct in *Streptomyces nodosus* exhibited lower hemolytic toxicity (Walmsley et al., 2017). In addition, highly water-soluble amphotericin B analogues were produced by the addition of extra sugar residues to amphotericin B (Caffrey et al., 2016). Recently, the discovery of C35deOAmB revealed that the antifungal activity was critical for binding to ergosterol, not for ion-channel formation (Gray et al., 2012), which generated toxicity-reduced amphotericin B derivatives (Cioffi et al., 2015; Muraglia et al., 2019). Overall, the engineering of polyene PKS to change the number of conjugated double bonds as well as post-PKS modifications for manipulation of the sugar and hydroxyl moieties is considered an important strategy for the generation of pharmacokinetically improved novel polyene derivatives (Bruheim et al., 2004; Gray et al., 2012; Caffrey et al., 2016; Walmsley et al., 2017; Muraglia et al., 2019).

NPP A1 is a disaccharide-containing polyene antifungal compound produced by a rare actinomycete, *Pseudonocardia autotrophica*, with an identical core macrolactone structure to nystatin A1 except for an additional *N*-acetyl-glucosamine (Figure 1; Lee et al., 2012). In the NPP A1 biosynthesis process, the macrolide backbone is synthesized by six type I PKSs, and the unique di-sugar moiety, mycosaminyl-(α1-4)-*N*-acetyl-glucosamine, is then attached by two glycosyltransferases-encoding *nppDI* and *nppY* and finally hydroxylated by P450 hydroxylase-encoding *nppL* (Kim et al., 2015). In terms of the biological activities, this polyene has superior water solubility and reduced hemolytic toxicity but shows slightly lower antifungal activity than nystatin A1 (Lee et al., 2012). Elucidation of the post-PKS modification steps of NPP A1 biosynthesis in *P. autotrophica* highlighted the potential to develop novel NPP A1 derivatives (Kim et al., 2015). Several studies were conducted to develop NPP A1 derivatives to enhance the antifungal activity, resulting in two analogues, NPP A2 and NPP B1. NPP A2 lacks a C10 hydroxyl group that was generated by the inactivation of the P450 hydroxylase-encoding *nppL* gene in *P. autotrophica* (Kim et al., 2016). NPP B1 was generated by changing its core macrolactone structure from tetraene to heptaene through site-specific, two amino acid substitutions in the putative NADPH-binding motif of the enoyl reductase domain in module 5 of the NPP A1 polyketide synthase NppC (Figure 1; Kim et al., 2018). Interestingly, both NPP A2 and NPP B1 exhibited higher antifungal activities than those of NPP A1.

In this study, another NPP A1 analogue called NPP B2 was developed, which was generated by the inactivation of the polyketide enoyl reductase (ER) domain in the fifth module as well as the P450 hydroxylase-encoding *nppL* gene in the NPP A1 producing *P. autotrophica* strain (Figure 1). To overcome the extremely low level of NPP B2 production because of its pathway refactoring, the 32-kb NPP-specific regulatory gene cluster was overexpressed in the NPP B2 production strain, followed by isolation and characterization of the biological activities of NPP B2.

## Materials and Methods

### Strains and Growth Conditions

*P. autotrophica* KCTC9441 purchased from the Korean Collection for Type Cultures was used for NPP production. The strain was grown routinely in ISP2 agar (malt extract 10 g, yeast extract 4 g, glucose 4 g, and agar 20 g/L) at 28°C for the sporulation and seed culture. YEME medium (yeast extract 3 g, peptone 5 g, malt extract 3 g, glucose 10 g, sucrose 340 g/L, and 5 mM MgCl_2_) was used to produce the NPP derivatives (Lee et al., 2012). *Candida albicans* ATCC 14053 was grown on YM medium (dextrose 10 g, peptone 5 g, yeast extract 3 g, Malt extract 3 g, and agar 20 g/L) at 30°C for 24 h. All *Escherichia coli* strains were incubated at 37°C in Luria-Bertani medium supplemented with the appropriate antibiotics where needed.

### Inactivation of NppL and Overexpression of NPP-Specific Regulatory Gene Cluster

A *nppL* gene inactivation cassette, including the upstream and downstream regions, was constructed by PCR amplification using the following primer pairs: upstream region, DELL_1F (5′-GAATTCCGTCCTGTACTCGTCGGT-3′) and DELL_1R (5′-CTGCAGTCA TGACGCGTCCTCCGT-3′) and downstream region, DELL_2F (5′-CTGCAGAC GCGGTCACGATGGCGC-3′) and DELL_2R (5′-AAGCTTACCTGGCCGAGCAGATGG-3′) (Kim et al., 2015). The amplified fragments were digested with *Hind*III-*EcoR*I and ligated into pKC1132. The recombinants were selected on LB medium containing apramycin. The *nppL* gene-inactivation plasmid was introduced into the chromosome of the NPP B1 production strain, *P. autotrophica* ER5 mutant.

To overexpress the NPP-specific regulatory genes, the previously constructed pNPPREG, encompassing the 32-kb NPP-specific regulatory gene cluster, was integrated into the chromosome of the B2 production strain (Han et al., 2019). The recombination conjugants were selected on ISP2 medium containing apramycin.

### Production and Purification of NPP Derivatives

NPP or its derivative production strains were inoculated in 300 ml of ISP2 medium containing the appropriate antibiotics at 30°C and 220 rpm for 72 h. The pre-cultures were added to 3 L YEME medium in a 5 L bioreactor for batch fermentation. After 48 h of cultivation, 150 g of Amberlite XAD16 resin (Sigma-Aldrich, USA) was added to the culture broth. After 24 h of resin addition, the mycelia and resin from the culture broth were separated and then extracted twice in 600 ml of *n*-butanol. The extract was concentrated using a vacuum evaporator, after which the concentrated extract was dissolved in methanol and loaded onto a column packed with a C18 reversed-phase silica gel (Daiso, Japan) along with methanol-water (30:70, v/v) to remove any residual sugar from the production media. The extracts with sugar removed were purified using a fraction collector (Interchim, France) on a gradient consisted of solvents A (water) and B (methanol): 30% B (v/v) (0–10 min) and 100% B (v/v) (100 min) at a flow rate of 20 ml/min. The fractions containing NPP or its derivatives with >80% purity were detected at 405 nm and analyzed by high performance liquid chromatography (HPLC). The column was equilibrated with 50% solvent A (0.05 M ammonium acetate, pH 6.5) and 50% solvent B (methanol); the flow rate was set to 1.0 ml/min using the following conditions: 0–3 min, 50–75% B; 3–30 min, 75–100% B; 30–33 min, 100–50% B; and 33–40 min, 50% B (Won et al., 2017).

### Liquid Chromatography-Mass Spectrometry/Mass Spectrometry Analysis

NPP B2 showing >80% purity was analyzed using A Triple TOF 5600 + (AB Sciex, USA) coupled with Ultimate3000 (Thermo Scientific, USA). Mass spectrometry was operated in both positive and negative ion modes over a mass range from 50 to 2,000 *m*/*z* using an electrospray ionization source. The settings were nitrogen gas for nebulization at 50 psi, heater gas pressure at 50 psi, curtain gas at 25 psi, temperature of 500°C, and an ion spray voltage at 5,500 V in positive ion mode and −4,500 V in negative ion mode. The optimized declustering potential (DP) and collision energy (CE) were set to 60 and 10 eV in positive ion mode, and to −60 and −10 eV in negative ion mode, respectively. A sweeping collision energy setting at 35/−35 ± 15 eV was applied for collision-induced dissociation (CID). Chromatographic conditions: solution A (0.1% formic acid in distilled water) and solution B (0.1% formic acid in acetonitrile) were used for elution and loaded onto Phenomenex Kinetex 1.7 μ C18 (2.1 mm × 150 mm, 1.7 μm). The flow rate was set to 0.4 ml/min using the following conditions: 0–1 min, 90% A; 1–5 min, 90–50% A; 5–18 min, 50–0% A; 18–25 min, 0% A; 25–27 min, 0–90% A; and 27–30 min, 90% A.

### RNA Analysis by Quantitative Real-Time Polymerase Chain Reaction

RNA was prepared using the RNeasy Mini Kit (Qiagen, Germany). cDNA conversion was carried out using a PrimeScript 1^ST^ strand cDNA Synthesis Kit (TaKaRa, Japan) according to the manufacturer’s instructions. Real-time PCR was performed using TaKaRa SYBR Premix Ex Taq (Perfect Real Time) with a Thermal Cycler Dice Real Time System Single (code TP850; TaKaRa, Japan). Supplementary Table S1 lists the primer pairs. The PCR conditions included activation for 10 min at 95°C, followed by 35 cycles of 30 s at 95°C, 30 s at 58°C, and 30 s at 72°C. The data were collected during each 72°C step, and melting curve analysis was performed at default settings ranging from 60 to 95°C. The relative level of amplified mRNA was normalized to the mRNA expression level of the housekeeping gene, *P. autotrophica hrdB*, which was amplified as an internal control using the primer pairs *hrdB*_F (5′-GCGGTGGAGA AGTTCGACTA-3′) and *hrdB*_R (5′-TTGATGACCTCGACCATGTG-3′) (Han et al., 2019).

### *In vitro* Assays for Biological Activities

For *in vitro* antifungal assay, we adapted to the Clinical and Laboratory Standards Institute document M27-A3 (Wayne, 2008). After *C. albicans* was cultured in YM medium at 30°C for 24 h, the cultured solution was diluted with YM medium until the OD value is 0.3 at 530 nm. A working suspension was made by a 1:2,000 dilution with RPMI-1640 broth media (with glutamine and phenol red, without bicarbonate, Sigma-Aldrich, USA), which resulted in 5.0 × 10^2^ to 2.5 × 10^3^ cells per μl. Ten microliters of the DMSO containing polyene antibiotics at various concentrations (3.125–1600 μg/ml) were added to the working suspension of 990 μl, and then, the mixtures were incubated at 30°C without shaking for 48 h. The colorimetric change of the mixture from red to yellow indicated the growth of *C. albicans.* The minimum inhibitory concentration (MIC) values were determined by measuring the minimum concentration that changed color to yellow. The experiment was performed in the duplicate.

For *in vitro* hemolysis assay, we adapted from a previously reported method (Nedal et al., 2007). Briefly, defibrinated horse blood was purchased from Kisan Biotech (South Korea). The polyene compounds were then prepared to the following concentrations with DMSO: 1–200 μg/ml. A 50 μl of each polyene solution was added to 450 μl of 2.5% defibrinated horse blood buffered with RBC buffer (10 mM NaH_2_PO_4_, 150 mM NaCl, 1 mM MgCl_2_, and pH 7.4), which resulted in a 1:10 dilution of each concentration of polyene. The samples were then incubated at 37°C for 30 min. After incubation, the samples were centrifuged at 10,000 ×*g* for 2 min. Next, 100 μl of the supernatant from each sample was added to a 96-well plate, after which the absorbance was read at 540 nm using a microplate reader (TECAN, Switzerland). The percentage hemolysis of each sample was defined as (Abs_sample_ − Abs_negative_/Abs_positive_ − Abs_negative_) × 100 (%) to calculate the minimum hemolysis concentration (MHC). The positive hemolysis sample was prepared by adding defibrinated horse blood 11.25 μl and DMSO 50 μl to distilled water 438.75 μl, and the negative hemolysis sample was prepared by adding defibrinated horse blood 11.25 μl and DMSO 50 μl to RBC buffer 438.75 μl.

## Results

### Construction of the NPP B2 Production Strain

**Figure 2:**
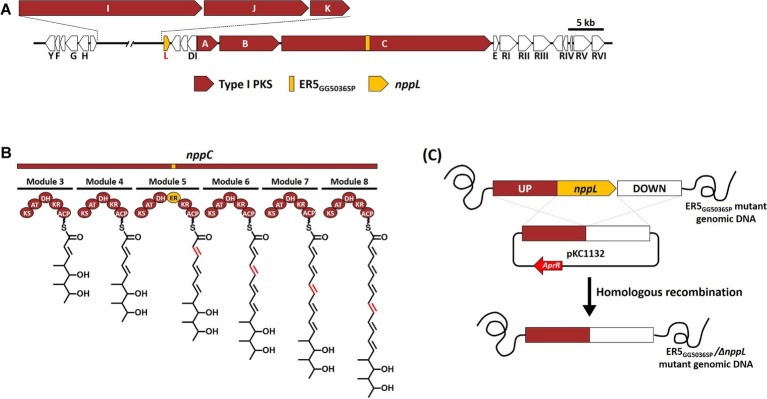
**(A)** Development of NPP B2 production strains in *P. autotrophica*. **(B)** Organization of modular polyketide synthases involved in mutated NppC by inactivation of enoyl reductase domain in module 5 (ER5). ACP, acyl carrier protein; AT, acyl-transferase; KS, ketosynthase; KR, ketoreductase; DH, dehydratase; ER, enoyl reductase. **(C)** Inactivation scheme of *nppL* gene in NPP B1 production strain (ER5 domain mutant).

An *in vitro* antifungal assay confirmed that the NPP B1, a heptaene version of NPP A1, showed much higher antifungal activity than the tetraene NPP A1 (Kim et al., 2018). Therefore, we tried to generate pharmacokinetically improved heptaene NPP B1 derivative through the engineering of post-PKS modification. Based on the previous results that characterization of the P450 hydroxylases, which were a region-specific hydroxylation such as AmphL (amphotericin), NysL (nystatin), and NppL (NPP) (Byrne et al., 2003; Volokhan et al., 2006; Kim et al., 2016), the NPP B1 production strain was engineered by inactivation of the *nppL* gene, which was involved in NPP C10 region-specific hydroxylation (Figures 2A,B; Supplementary Figure S1). Inactivation of the *nppL* gene was performed successfully by homologous recombination using the pKC1132 *Streptomyces* suicide vector system, and the mutation was verified genetically by PCR product sequencing analysis (Figure 2C; Supplementary Figure S2). LC-MS analysis of purified NPP B2 contained a signal at *m*/*z* 1111.5807 for [C_53_H_85_N_2_O_22_]^+^ (calculated mass of NPP B2 is 1110.57) confirming that NPP B2 had been produced, as expected in the constructed mutant strain (Supplementary Figure S3).

### Stimulation of NPP B2 Production Through Overexpression of the NPP Pathway-Specific Regulatory Genes

**Figure 3:**
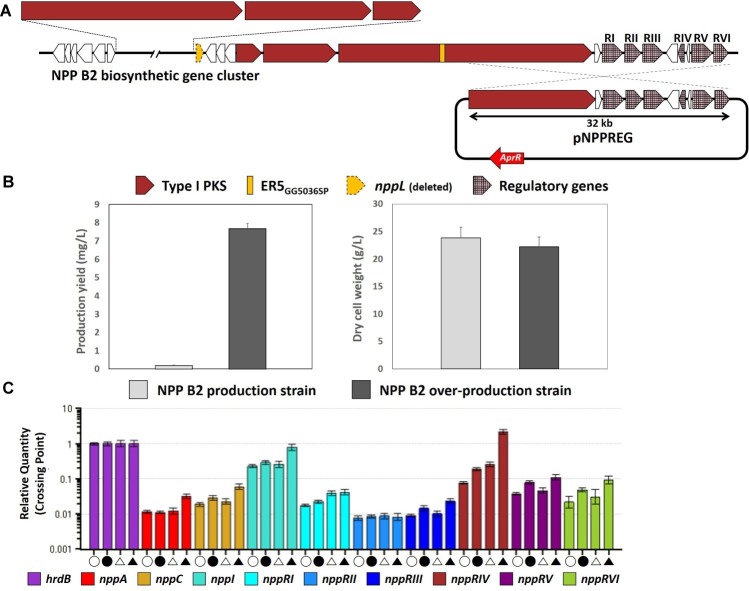
**(A)** Overexpression scheme of NPP-specific regulatory gene cluster in NPP B2 production strain. **(B)** Comparison of NPP B2 production yields with newly constructed NPP B2 over-production strain after 72 h culture. **(C)** Transcript analysis of NPP B2 production strains by qRT-PCR. Open circle, transcripts from the NPP B2 production strain at 24 h; closed circle, transcripts from the NPP B2 production strain at 48 h; open triangle, transcripts from the NPP B2 over-production mutant at 24 h; closed triangle, transcripts from the NPP B2 over-production mutant at 48 h; house-keeping gene, *hrdB* (light purple); PKS genes, *nppA* (red), *nppC* (yellow), and *nppI* (emerald green); regulatory genes related to NPP B2 biosynthesis, *nppRI* (sky blue), *nppRII* (blue), *nppRIII* (dark blue), *nppRIV* (brown), *nppRV* (dark purple), and *nppRVI* (light green). All transcript measurements were performed in duplicate.

Although the NPP B2 production strain was developed successfully by inactivation of the ER domain of module 5 followed by the P450 hydroxylase-encoding *nppL* gene from the NPP A1 production strain, the NPP B2 production level was reduced approximately 40-fold to 0.2 mg/L compared to the NPP A1 production level in the *P. autotrophica* wild type. Among the attempts to increase the production of NPP derivatives, a strategy was conducted to increase the production level of NPP B2 through overexpression of the NPP pathway-specific regulatory genes. In previous studies, pNPPREG was constructed by cloning a 32-kb right-hand portion of BGC containing the six NPP-specific regulatory genes (*nppRI*–*nppRVI*) into a *Streptomyces* artificial chromosomal vector pSBAC (Han et al., 2019). To stimulate NPP B2 production, pNPPREG containing the entire regulatory genes was integrated into the chromosome of the NPP B2 production strain (Figure 3A). As a result, the level of NPP B2 production was increased significantly (approximately 39-fold to 7.67 mg/L) (Figure 3B).

The transcription levels of the NPP B2 biosynthetic genes were analyzed to further understand the molecular basis responsible for enhanced NPP B2 production in the engineered NPP B2 over-production strain. qRT-PCR analysis revealed increased transcription of the PKS genes, such as *nppA* and *nppC*, in the NPP B2 over-production strain compared to the parental strain (Figure 3C). As expected, the transcription levels of all five NPP-pathway specific regulatory genes, except *nppRII,* were also stimulated (Figure 3C), suggesting that the enhanced transcription level of the refactored NPP B2 biosynthetic pathway genes is critical for the titer improvement.

### *In vitro* Antifungal Activity and Hemolytic Toxicity

Based on successful NPP B2 yield improvement, the purified NPP B2 was evaluated for both *in vitro* antifungal activity and the hemolytic toxicity. Minimum inhibitory concentration (MIC) evaluation assays of *in vitro* antifungal activity using the colorimetric change in the RPMI-1640 media containing *C. albicans* were employed (Wayne, 2008). The MIC value of NPP B2 against *C. albicans* (1.0 μg/ml) was slightly higher than amphotericin B (0.25 μg/ml) and NPP B1 (0.5 μg/ml) (Table 1; Supplementary Figure S4). Interestingly, however, *in vitro* hemolytic toxicity evaluation, NPP B2 exhibited much reduced hemolytic toxicity than its parental NPP B1. The minimum hemolytic concentration (MHC) value for NPP B2 was measured as 80.18 μg/ml, whereas the MHC value of amphotericin B was 3.26 μg/ml under the conditions tested, indicating that the *in vitro* toxicity of NPP B2 was approximately 25-fold lower than that of amphotericin B (Table 1; Supplementary Figure S5). Moreover, the *in vitro* toxicity was 4.5-fold lower than the MHC value of NPP B1 (17.72 μg/ml). These results suggest that the absence of the hydroxyl moiety at the C10 position of NPP B2 could play an important role in controlling both antifungal acidity and hemolytic toxicity.

**Table 1:** *In vitro* antifungal activity and toxicity of polyene macrolides.

	Amphotericin B	NPP B1	NPP B2
**Antifungal activity (MIC, μg/ml)^a^**			
*Candida albicans* ATCC 14053	0.25	0.5	1.0
**Hemolytic toxicity (MHC, μg/ml)^b^**			
	3.26 ± 0.259	17.71 ± 1.235 (5.4-fold)	80.18 ± 1.168 (25-fold)

a *MIC, minimum inhibitory concentration (values resulting in no visible growth of C. albicans)*.

b *MHC, minimum hemolytic concentration (values causing 90% hemolysis against horse blood cells ± percentage standard deviation)*.

## Discussion

The genome mining approach of rare actinomycetes followed by the activation of its cryptic biosynthetic gene cluster (BGC) has become an attractive strategy to screen and develop novel bioactive compounds (Choi et al., 2018). The *P. autotrophica* strain described here was classified originally as a polyene non-production strain, which was later proven to be a producer of a novel di-saccharide-containing NPP A1 through culture optimization and whole genome sequencing (Lee et al., 2012). Although an NPP A1 BGC refactoring strategy generated a C10-deoxy NPP A1 (named NPP A2) and a heptaene version of NPP A1 (named NPP B1), their extremely low titers were major hurdles for further characterization of their biological activities.

Previously, several strategies, including overexpression of the pathway-specific regulatory gene, deletion of the global antibiotic downregulator, *in situ* screening of random mutants, co-culture system, and cultivation with xenobiotics, all failed to improve NPP B1 production (Kim et al., 2018). Recently, the entire cluster containing all six NPP pathway-specific genes in a pSBAC system followed by its re-integration into the *P. autotrophica* chromosome led to a significant increase in the NPP B1 titer (Han et al., 2019).

In this study, another novel derivative, called the NPP B2 production strain, was first generated by site-specific inactivation of the *nppL* gene in the NPP B1 production of the *P*. *autotrophica* mutant strain. C10-deoxy NPP B1 (NPP B2) was also generated based on a previous report that the C8-deoxy amphotericin produced by the inactivation of *amphL* in *S. nodosus* and C10-deoxy nystatin by the inactivation of *nysL* in *Streptomyces noursei* (Byrne et al., 2003; Volokhan et al., 2006). Owing to the extremely low titer of NPP B2, however, its biological characterization could not be pursued without strain improvement. Through the chromosomal integration of all six NPP pathway-specific genes in the NPP B2 production strain, its titer was improved significantly (approximately 39-fold), which was sufficient to proceed for further biological assays, including antifungal and hemolytic toxicity assays.

This paper described for the first time the *in vitro* biological activities of NPP B2, a novel heptaene version of the NPP derivative. Although the *in vitro* antifungal activity of NPP B2 was higher than that of tetraene-type polyenes, such as nystatin A1, NPP A1, and A2, it showed slightly lower antifungal activity than other heptaene-type polyenes, including amphotericin B and NPP B1. Interestingly, the *in vitro* hemolytic activity of NPP B2 was approximately 25 times lower than those of amphotericin B and NPP B1, suggesting that the hydroxyl moiety at the C10 position of NPP could play a critical role in controlling both the antifungal activity and the hemolytic toxicity, probably by affecting its binding affinity to cholesterol and ergosterol. Overall, these results suggest that the combination of rational BGC refactoring and its genetic strain improvement approach is an efficient strategy to stimulate the production of an extremely low-level metabolite, such as NPP B2 in a rare actinomycetes strain.

## Data Availability Statement

The datasets generated for this study can be found in the EU108007.

## Author Contributions

H-SP, S-SC, and E-SK designed the experiments. H-SP, H-JK, and C-YH performed the experiments. H-SP, H-JN, and E-SK wrote the manuscript.

### Conflict of Interest

The authors declare that the research was conducted in the absence of any commercial or financial relationships that could be construed as a potential conflict of interest.
