# Supplementary material for: Stimulated Biosynthesis of an C10-Deoxy Heptaene NPP B2 via Regulatory Genes Overexpression in Pseudonocardia autotrophica
Source: Front Microbiol. 2020 Jan 24;11:19. doi: 10.3389/fmicb.2020.00019 (PMC6993583; doi:10.3389/fmicb.2020.00019)
Supplement: Supplementary file 1 [file Data_Sheet_1.docx]

**Stimulated biosynthesis of an C10-deoxy heptaene NPP B2 via regulatory genes overexpression in *Pseudonocardia autotrophica***

**Heung-Soon Park, Hye-Jin Kim, Chi-Young Han, Hee-Ju Nah, Si-Sun Choi, and Eung-Soo Kim^*^**

Department of Biological Engineering, Inha University, Incheon 22212, Republic of Korea

^*^Corresponding authors:

E-mail: eungsoo@inha.ac.kr; Phone: +82-32-860-8318; Fax: +82-32-865-4046

Running title: *Pseudonocardia* NPP B2 regulatory genes overexpression

Key words: *Pseudonocardia autotrophica,* polyene, regulatory genes, hemolytic toxicity, antifungal activity

**Supplementary Figure S1.** Comparison of HPLC Analysis Results of NPP B1 and NPP B2 at 405nm.

NPP B1

NPP B2

**Supplementary Figure S2.** Deletion of *nppL* from NPP B1 production strain. Geneticconfirmation using the following primer pair: Check F (5’-CTCAACAGCGCGCGGATGTC -3’) and Check R (5’- ACCTGGTGCGGCTGGAGATG -3’).


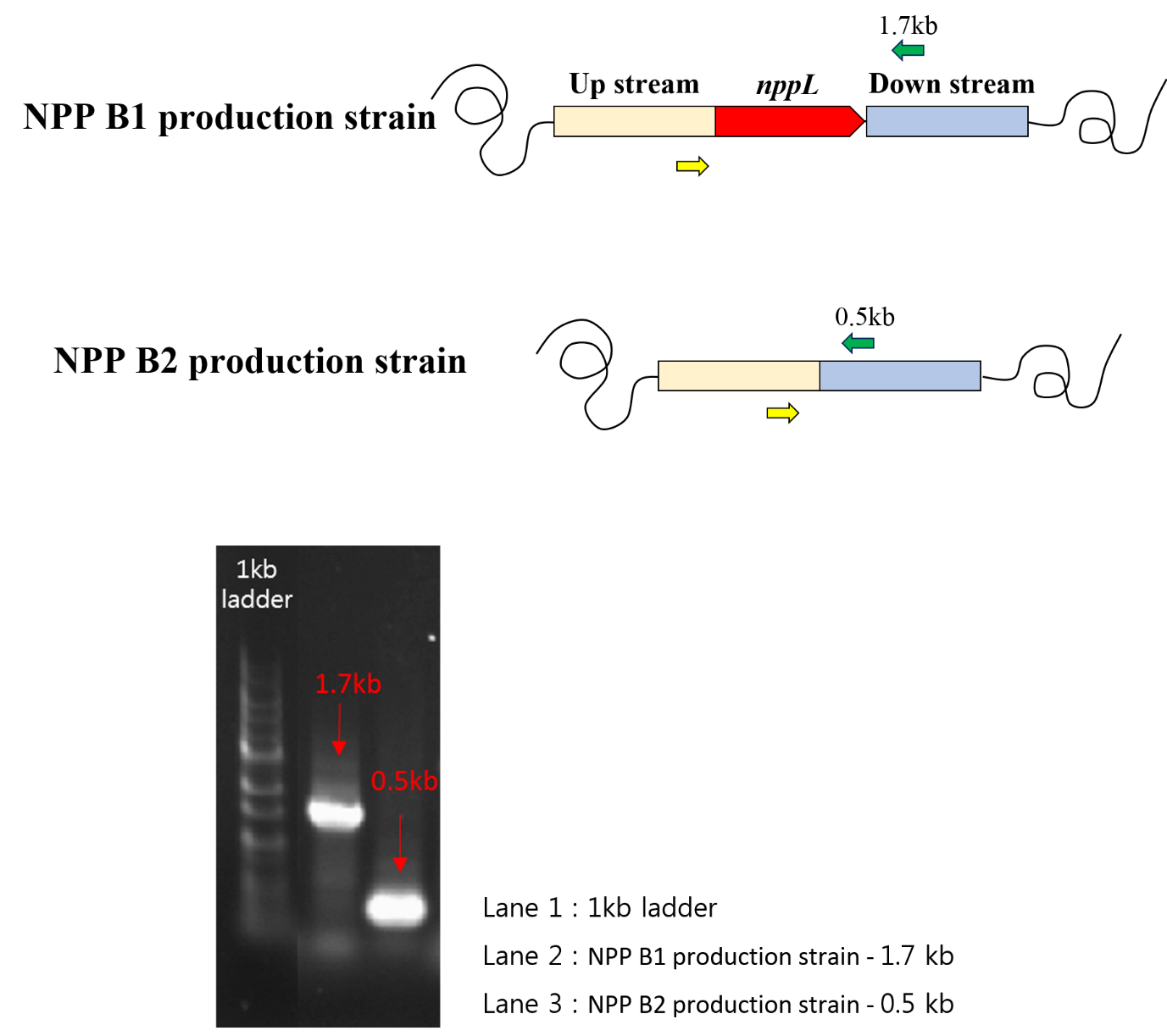

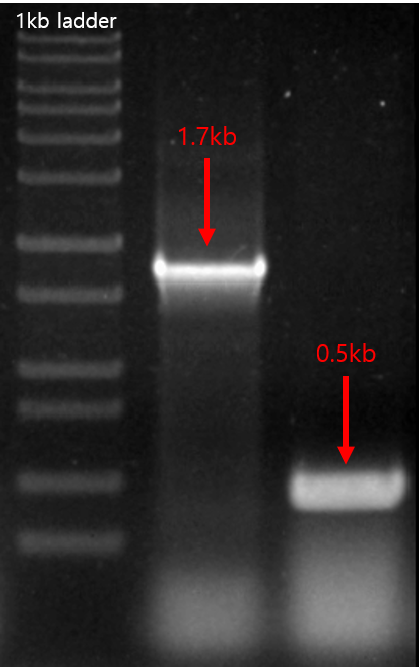


**Supplementary Figure S3.** High Resolution Mass Spectrometry (HRMS) in positive ion mode to determine the production of NPP B2.

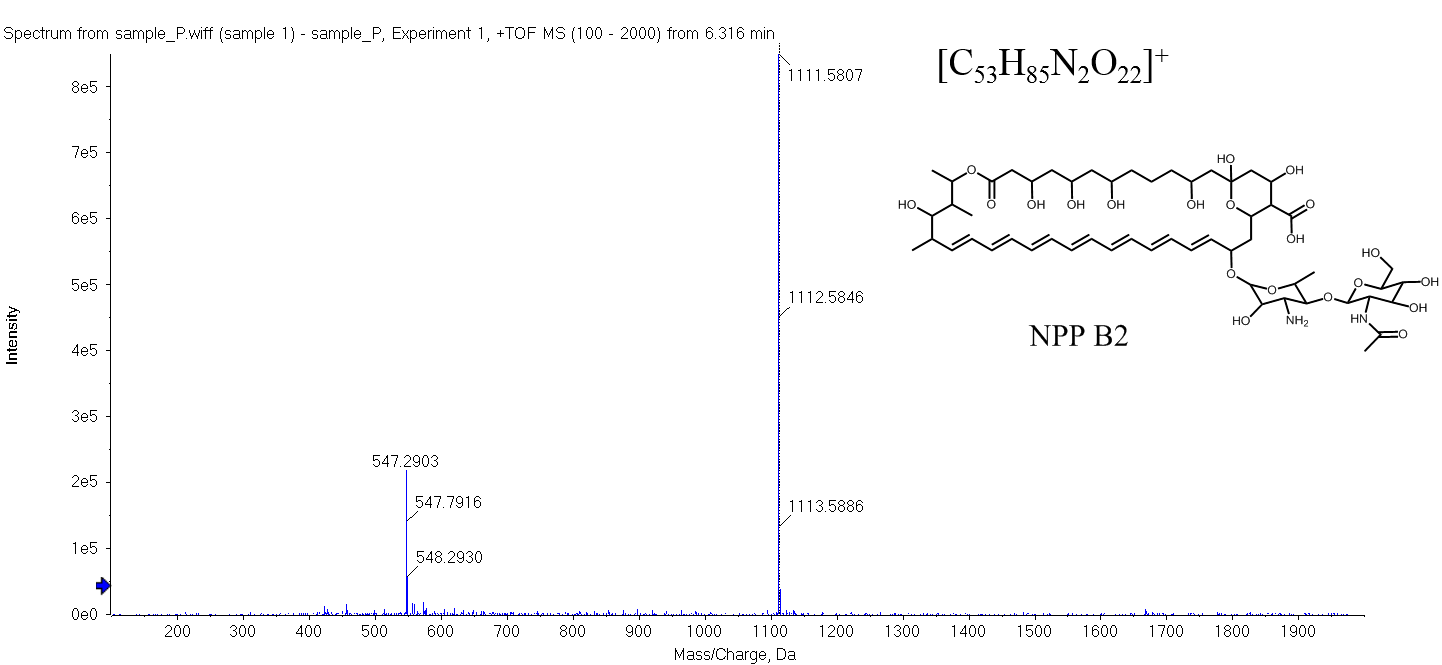


**Supplementary Figure S4.** *In vitro* antifungal activity of polyene macrolides using the RPMI-1640. 10 μl of the DMSO containing polyene antibiotics at various concentrations (3.125 to 1600 μg/ml) were added to the working suspension of 990 μl and then the mixtures were incubated at 30 °C without shaking for 48 hr. The minimum inhibitory concentration (MIC) values was determined by measuring the minimum concentration that changed color to yellow.


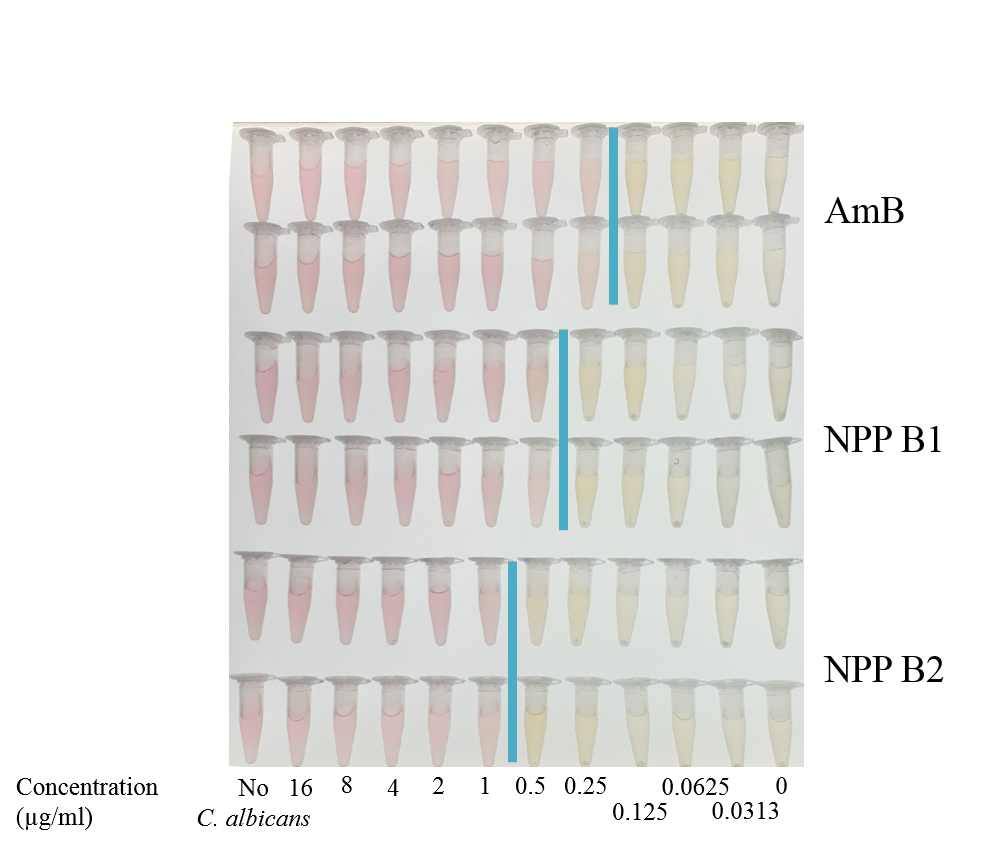


**Supplementary Figure S5.** *In vitro* hemolytic toxicity of polyene macrolides.

**
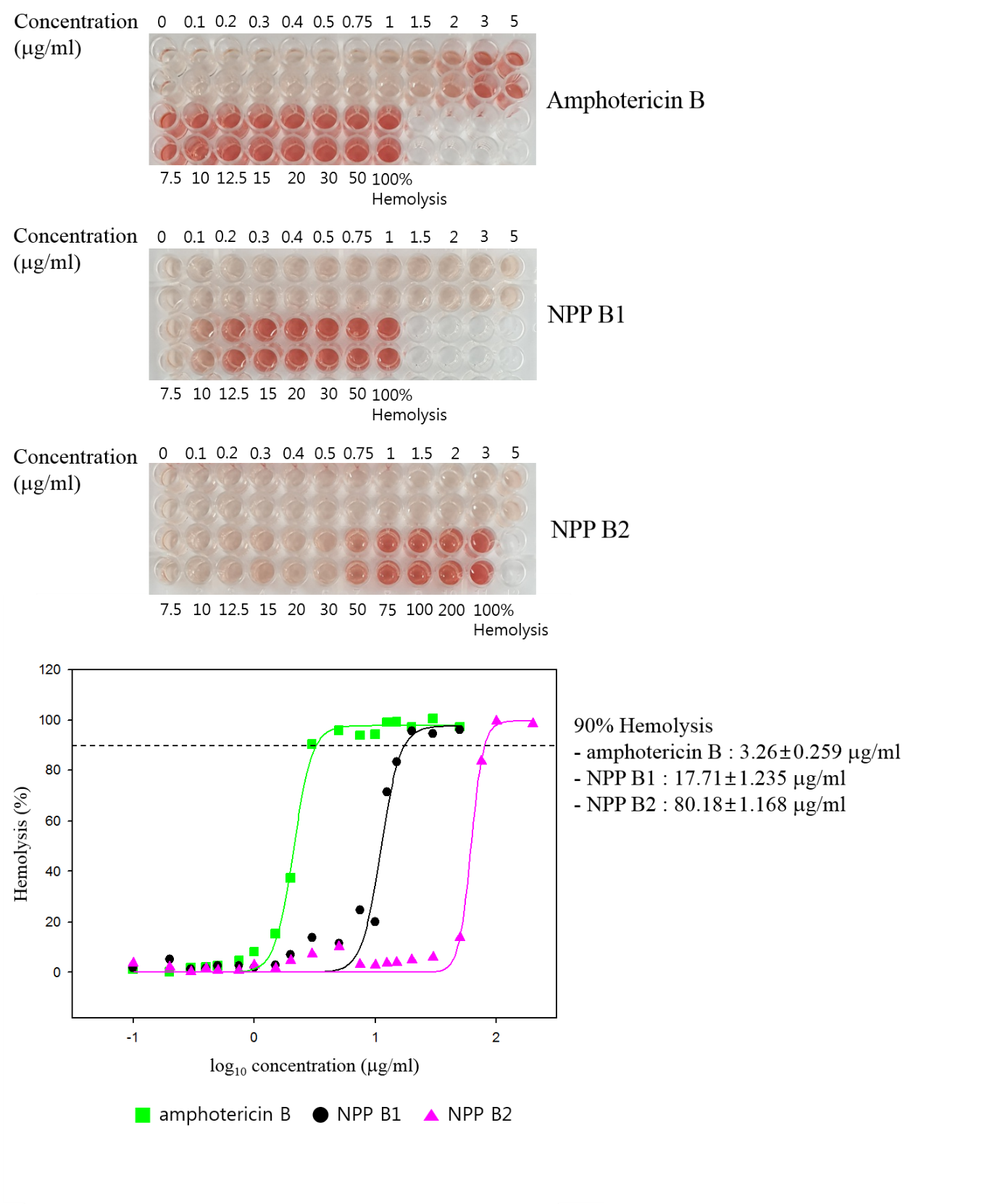
**

**Supplementary Table 1:** List of qRT-PCR primers

| **Primer** | **Primer sequence, 5’→3’** |
| --- | --- |
| *nppA*_F | GGCCACTCCATCGGTGAGAT |
| *nppA*_R | GTAGCGCCTGCATGAGTGTG |
| *nppC*_F | GAGTACCGGAACCGGCTCAA |
| *nppC*_R | GGGAAGTCGAACAGCAGGGT |
| *nppI*_F | TCCGCATCCTCACCGAGAAC |
| *nppI*_R | CGATGATGGTGTGGGCGTTG |
| *nppRI*_F | CGGACCGGACAACCTCTACC |
| *nppRI*_R | CAGCAGGAACCCGAGGAAGG |
| *nppRII*_F | GACGTCCTGCACGAGTGGT |
| *nppRII*_R | CACTGTCCCGTGTCCTCGTC |
| *nppRIII*_F | CTGGAGTGCCTGCTCGACTG |
| *nppRIII*_R | CTCCTCGTCGGCCCATTCC |
| *nppRIV*_F | GCCTCCACCCTGTTCCTCAG |
| *nppRIV*_R | CCACGAACCCAGCTCGAAGA |
| *nppRV*_F | GGCCTGACCAACCACGAGAT |
| *nppRV*_R | AGGCGATACGATCGGAACCA |
| *nppRVI*_F | CCTGGCGGTGGAACTACCTG |
| *nppRVI*_R | ACGATCCCGACAGCCATCAG |
